# Energy-based analog neural network framework

**DOI:** 10.3389/fncom.2023.1114651

**Published:** 2023-03-03

**Authors:** Mohamed Watfa, Alberto Garcia-Ortiz, Gilles Sassatelli

**Affiliations:** ^1^LIRMM, University of Montpellier, CNRS, Montpellier, France; ^2^ITEM, University of Bremen, Bremen, Germany

**Keywords:** neural networks, energy-based models, equilibrium propagation, framework, analog, mixed-signal, SPICE

## Abstract

Over the past decade a body of work has emerged and shown the disruptive potential of neuromorphic systems across a broad range of studies, often combining novel machine learning models and nanotechnologies. Still, the scope of investigations often remains limited to simple problems since the process of building, training, and evaluating mixed-signal neural models is slow and laborious. In this paper, we introduce an open-source framework, called EBANA, that provides a unified, modularized, and extensible infrastructure, similar to conventional machine learning pipelines, for building and validating analog neural networks (ANNs). It uses Python as interface language with a syntax similar to Keras, while hiding the complexity of the underlying analog simulations. It already includes the most common building blocks and maintains sufficient modularity and extensibility to easily incorporate new concepts, electrical, and technological models. These features make EBANA suitable for researchers and practitioners to experiment with different design topologies and explore the various tradeoffs that exist in the design space. We illustrate the framework capabilities by elaborating on the increasingly popular Energy-Based Models (EBMs), used in conjunction with the local Equilibrium Propagation (EP) training algorithm. Our experiments cover 3 datasets having up to 60,000 entries and explore network topologies generating circuits in excess of 1,000 electrical nodes that can be extensively benchmarked with ease and in reasonable time thanks to the native EBANA parallelization capability.

## 1. Introduction

The past decade has seen a remarkable series of advances in deep learning (DL) approaches based on artificial neural networks (ANN). In the drive toward better accuracy, the complexity, and resource utilization of state-of-the-art (SOTA) models have been increasing at such an astounding rate that training and deploying these models often require computational and energy resources that lie outside the reach of most resource-constrained edge environments (Bianco et al., 2018). As a result, most of the training and processing has been done in data centers that require access to the cloud. However, energy cost, scalability, latency, data privacy, etc., pose serious challenges to existing cloud computing. Alternatively, edge computing has emerged as an attractive possibility (Wang et al., 2020).

The high computational and power demands of DL are driven by two key factors. The first is the efficiency of the DL algorithms. Current SOTA models require multiply-and-accumulate operations (MACs) that number in the billions. For example, VGGNet (Simonyan and Zisserman, 2015), a model that enabled significant accuracy improvements in the ImageNet challenge, required 138M parameters and 15.5G MACs. These numbers are even higher for current SOTA models (Sevilla et al., 2022).

The second component of the power equation is tied to the hardware architecture on which the DL workloads are executed. Machine learning and other data intensive workloads are fundamentally limited by computing systems based on the von Neumann architecture, which has separate memory and processing units, and thus wastes a lot of energy in memory access and data movement. For instance, to support its 724M MACs, AlexNet requires nearly 3 billion DRAM accesses, where fetching data from off-chip DRAM costs 200 × more energy compared to fetching data from the register file (Sze et al., 2017).

With energy-efficiency being a primary concern, the success of bringing intelligence to the edge is pivoted on innovative circuits and hardware that simultaneously take into account the computation and communication that are required. Consequently, recent hardware architectures for DL show an evolution toward “in/near-memory” computing with the goal of reducing data movement as much as possible. One category of such architectures, the so-called Processing-In-Memory (PIM), consists in removing the necessity of moving data to the processing units by performing the computations inside the memory. This approach is commonly implemented by exploiting the analog characteristics of emerging non-volatile memories (NVM) such as ReRAM crossbars, though it is also possible to leverage mature CMOS-based technologies (Kim et al., 2017). Furthermore, as ANN inference is inherently resilient to noise, this opens the opportunity to embrace analog computing, which can be much more efficient than digital especially in the low SNR (signal-to-noise ratio) regime (Murmann et al., 2015). This work targets this class of ANNs.

Due to the highly demanding device and circuit requirements for accurate neural network training (Gokmen and Vlasov, 2016), most mixed-signal implementations are inference-only. While the optimal implementation of the memory devices is an on-going challenge, there is an opportunity to simplify the circuit requirements by considering learning algorithms that are well-matched with the underlying hardware. One such algorithm is the Equilibrium Propagation (EP) algorithm that leverages the fact that the equilibrium point of a circuit corresponds to the minimization of an abstract energy function (Scellier and Bengio, 2017), whose definition is discussed in Section 2. By allowing the bidirectional flow of signals, the EP method forgoes the need for a dedicated circuit during the backward phase of training, while also keeping the overhead of the periphery circuit that supports it to a minimum as there is no need for analog-to-digital converters between layers.

Given the growing rate of machine learning workloads, it is of paramount importance to have a framework that is capable of performing a comprehensive comparison across different accelerator designs and identify those that are most suitable for performing a particular ML task. Thanks to ML frameworks such as Google's Tensorflow and Keras, the ease of creating and training models is far less daunting than it was in the past. While training an analog neural network with EP could in theory be possible in Tensorflow, there are three major difficulties:

- First, the current-voltage (I-V) characteristic of each circuit element has to be completely defined. This also calls for the implementation of a non-linear equation solver.
- Second, the network layers have to be designed in such a way that they can influence each other in both directions. Without the loading effect, the model will fail to learn.
- Finally, implementing procedures that involve iterative updates, like differential equations, within automatic differentiation libraries like Tensorflow, would mean that we need to store all the temporary iterates created during this solution for each time step. This requires storing a great deal of information in memory. As will be explained later, when implemented on analog circuits, the EP method requires the data points at only two time steps.

Based on the above motivations, this work introduces an exploratory framework called EBANA (Energy-Based ANAlog neural networks), built in the spirit of Keras^1^ with two goals in mind: ease-of-use and flexibility. By hiding the complexity inherent to machine learning and analog electronics behind a simple and intuitive API, the framework facilitates experimentation with different network topologies and the exploration of the various trade-offs that exist in the design space.

In the relatively few studies that strive to understand the inner workings of the EP algorithm on analog hardware, we observe several cases where our framework could prove immediately beneficial. In Kiraz et al. (2022), the authors studied the impacts of the learning rate and the scaling factor of the feedback current on the algorithm convergence. Their experiments were carried out on a simple two-input-one-output circuit, and, therefore, it is not clear whether their results generalize to more complex circuits. The size of the network in our framework is limited only by the underlying SPICE simulator, thus facilitating much more comprehensive studies. In Foroushani et al. (2020), the authors built a circuit based on the continuous Hopfield model to learn the XOR circuit. The modularity and graph-based data structure of our framework can easily accommodate new analog blocks and topologies, making it easy to study their effect on accuracy, power estimation, etc., as the network size grows.

Although research on EBM-based ANN accelerators is still in its early stages, a substantial amount of work has been done on non-EBM-based accelerators. Most of these accelerators are designed for inference only, as on-chip training has proven to be challenging (Krestinskaya et al., 2018). To achieve speed and energy savings, these accelerators embed the computations inside memory elements such as emerging non-volatile memory (Li et al., 2015; Hu et al., 2016; Shafiee et al., 2016; Gokmen et al., 2018), floating-gate transistors (Agarwal et al., 2019; Park et al., 2019), or volatile capacitive memories (Boser et al., 1991; Bankman et al., 2019). For a more comprehensive overview of ANN architectures, the reader is referred to (Xiao et al., 2020).

This paper is organized as follows. In Section 2, we give a very brief introduction into energy based learning, and explain why it is a natural fit for analog systems. In Section 3, we provide an overview of the internals of our API, and illustrate with an example how quickly and easily models can be created. In Section 4, we validate our framework by training an analog circuit on a non-trivial ML task, evaluate the performance, and show how the framework can be extended. Finally, we discuss the conclusions and further work.

In this work, we expand upon our previous introduction of the EBANA framework in Watfa et al. (2022) by elaborating on the relationship between energy-based models and electrical circuits. Specifically, we demonstrate how the energy function can be shaped and modified by the learning process and examine the impact of various parameters on the learning capacity of the analog circuit. Additionally, we discuss the potential for interfacing an analog neural network based on the EP algorithm with one based on the backpropagation algorithm in a mixed-mode design.

## 2. Energy based learning

The main goal of deep learning or statistical modeling is to find the dependencies between variables. Energy Based Models (EBMs) encode these dependencies in the form of an energy function *E* that assigns low energies to correct configurations and high energies to incorrect configurations. However, unlike statistical models which must be properly normalized, EBMs have no such requirements (LeCun et al., 2006), and, as such, can be applied to a wider set of problems.

Two aspects must be considered when training EBMs. The first is finding an energy function that is rich enough to model the dependency between the input and output. This is usually tied to the architecture of the network. The second is shaping the energy function so that the desired input-output combinations have lower energy than all other (undesired) values. In the following sections, we consider one example of such a method, explain how it works, and discuss how it can be used to train analog neural networks.

### 2.1. An alternative to backpropagation

The success of deep neural networks can be attributed to the backpropagation (BP) algorithm, which exploits the chain rule of derivatives to compute updates for the parameters in the network during learning. In spite of its success, BP poses a few difficulties for implementation in hardware. The requirement for different circuits in both phases of training is one of the core issues that the EP learning framework sets out to address (Scellier and Bengio, 2017). It involves only local computations while leveraging the dynamics of energy-based physical systems. It has been used to train Spiking Neural Networks (Martin et al., 2021) and in the bidirectional learning of Recurrent Neural Networks (Laborieux et al., 2020).

**Figure 1:**
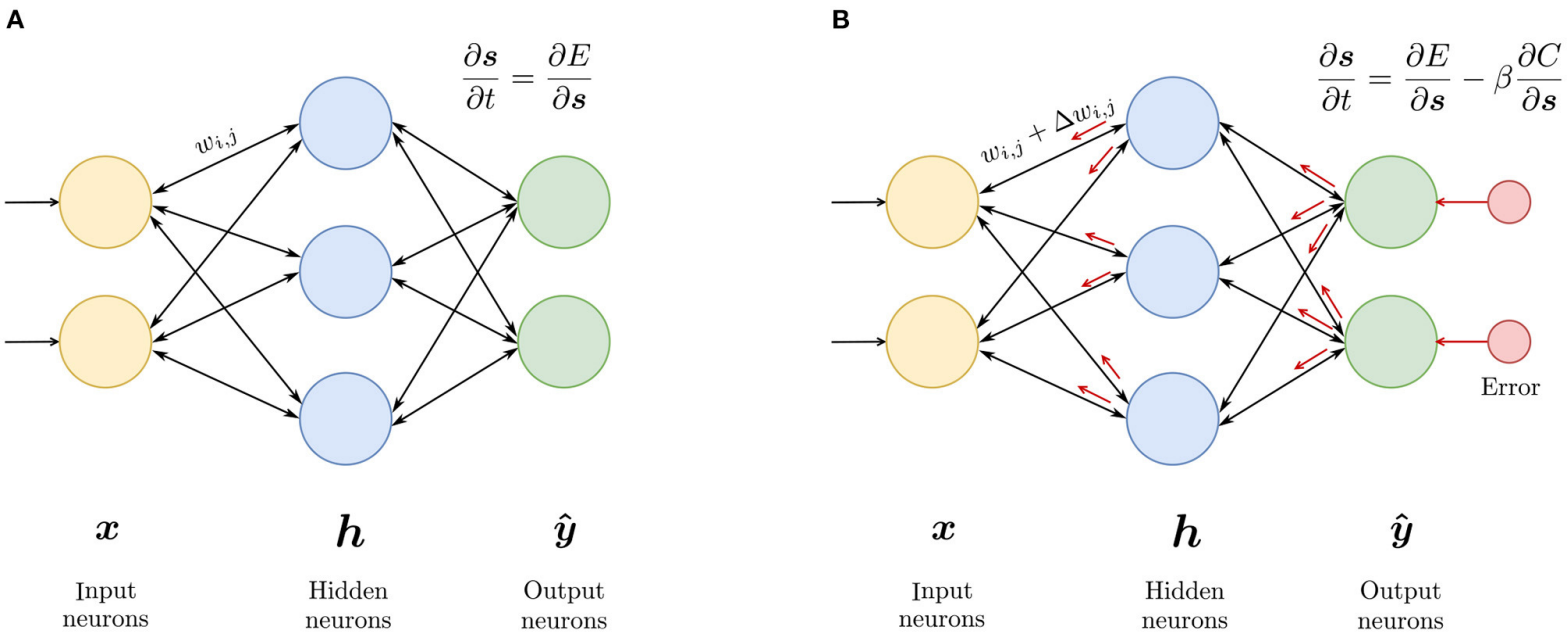
Equilibrium propagation algorithm: **(A)** In the *free* phase, the input is presented to the network and the network settles in an equilibrium state. **(B)** In the *nudging* phase, an error signal (depicted in red) is introduced at the output, forcing the network to settle in nearby equilibrium state, having a slightly lower energy than the *free* equilibrium state. The parameters are updated based on these two states.

The EP algorithm is a contrastive learning method in which the gradient of the loss function is defined as the difference between the equilibrium state energies of two different phases of the network. The two phases are as follows. In the *free* phase, the input is presented to the network and the network is allowed to settle into a *free* equilibrium state, thereby minimizing its energy. Once equilibrium is reached, inference result is available at the output neurons. In the second, *nudging* phase, an error is introduced to the output neurons, and the network settles into a *weakly-clamped* equilibrium state, which is closer to the desired state than the *free* equilibrium state. The parameters of the network are then updated based on these two equilibrium states. The idea is depicted in Figure 1.

### 2.2. Constructing the energy function

Supervised learning in a neural network is driven by the optimization of an error function of the output. A common objective is the minimization of mean squared error (MSE) or the cross-entropy of the network's output and the target output. However, in energy-based models the optimization objective is not a function of the output, but some scalar energy function of the entire network state.

The design of the energy *E* can be inspired from physics or hand-crafted based on the network architecture. An early example of EMBs is the Hopfield network and its stochastic variant, the Restricted Boltzmann Machine (Hinton, 2012). In these networks, the energy function is constructed by observing that a neuron only flips when the state of the neuron is opposite that of the field. The energy function is defined as the negative sum of the output of all the neurons, a number bounded by the parameters of the network. As the neurons flip, the overall energy of the system decreases until a configuration is reached that corresponds to the minimum of the energy function. The energy function of the RBM is presented below.


(1) $$\begin{array}{l} E\left(v,h\right)=-b^{T}v-c^{T}h-v^{T}Wh \end{array}$$

where ***θ*** = (***b***, ***c***, ***W***) are the real-valued parameters of the model. ***b*** and ***c*** are the bias vectors, and ***W*** is the weight matrix. The parameters represent the preference of the model for a particular value of ***v*** or ***h***.

Despite being an energy-based model, the RBM is trained using maximum-likelihood estimation (MLE) (Hinton, 2012), a standard method for training probabilistic models. The basic idea is to find the parameters of the network that maximize the likelihood of the dataset. This is a very slow and computationally expensive process, especially when the dimensionality of the dataset is high, as it requires sampling from the joint distribution of ***v*** and ***h***. The EP algorithm is able to avoid this by introducing a cost function to the energy function that nudges the system toward a state that reduces the cost value.

In the EP algorithm, the state ***s*** of the system is governed by the network energy function


(2) $$\begin{array}{l} F\left(\theta ,x,y,\beta ,s\right)=E\left(\theta ,x,s\right)+\beta \cdot C\left(\theta ,x,y,s\right), \end{array}$$

where ***θ*** = (***W***, ***b***) are the network parameters, ***x*** is the input to the network, ***y*** is the target output, and ***s*** = {***h***, ***ŷ***} is the collection of neuron states, comprised of the hidden and output neurons, respectively.

The total energy function *F* is composed of two sub-parts: the internal energy *E*, which is a measure of the interaction of the neurons in the absence of any external force, and the external energy or cost function *C*, modulated by the influence parameter β. The states are gradually updated over time to minimize the overall energy. The introduction of the cost function to the energy function is one of the main features that distinguishes the EP algorithm from other EBM-based algorithms.

### 2.3. Equilibrium propagation algorithm

Given a training example (***x***^(*i*)^, ***y***^(*i*)^) and ***θ*** in the absence of an external potential (β = 0), the system reaches a state $s^{0}=s_{0}\left(\theta ,x\right)$ that minimizes the internal energy *E*(***θ***, ***x***, ***s***). The cost function *C*(***θ***, ***x***, ***y***, ***s***) evaluates the quality of ***s***^0^ in mapping ***x***^(*i*)^ to ***y***^(*i*)^. If ***s***^0^ isn't adequate, a force proportional to $\frac{\partial C}{\partial \hat{y}}$ is applied to drive the output units toward their target, moving the system to a nearby state $s^{\beta }=s_{\beta }\left(\theta ,x,y\right)$ that has a lower prediction error. As opposed to RBMs where the output units are clamped to the desired values during the second phase of training, the output units are driven to the desired values in the EP algorithm, hence the term *weakly-clamped*. The perturbation at the outputs propagates across the hidden layers, causing the network to relax at a nearby state ***s***^β^, which is better than ***s***^0^ in terms of the prediction error. This corresponds to “pushing down” the energy of ***s***^β^, and “pulling up” the energy of ***s***^0^. A demonstration of this is presented in the next section.

The EP training algorithm is presented in Algorithm 1. Equation (3) shows how we can update the parameters of the network between the two phases. It is an approximation of the derivative of the loss function with respect to β (hence the $\frac{1}{\beta }$ term). For the interest of brevity, the reader is directed to the source material (Scellier and Bengio, 2017) for a detailed derivation of the equation.

**Algorithm 1:** Equilibrium propagation.

1. Fix the inputs and allow the system to settle in ***s***^0^ that corresponds to the local minimum of *E*(***θ***, ***x***, ***s***) or *F*(***θ***, ***x***, ***y***, 0, ***s***). Collect $\frac{\partial F}{\partial \theta }\left(\theta ,x,y,0,s^{0}\right)$. This is the **free phase**.
2. With the input still fixed, nudge the output units toward their target values. Allow the system to settle in a new but nearby fixed point ***s***^β^ that corresponds to slightly smaller prediction error. Collect $\frac{\partial F}{\partial \theta }\left(\theta ,x,y,\beta ,s^{\beta }\right)$. This is the **nudging phase**.
3. Update the parameter ***θ*** according to
(3) $$\begin{array}{l} \Delta \theta \propto -\frac{1}{\beta }\left(\frac{\partial F}{\partial \theta }\left(\theta ,x,y,\beta ,s^{\beta }\right)-\frac{\partial F}{\partial \theta }\left(\theta ,x,y,0,s^{0}\right)\right).\;\left(3\right) \end{array}$$

Compared to backpropagation, there are two important differences that make this approach especially attractive for implementation in hardware: First, propagating the errors toward the input does not require a special computational circuit (which is the case for backpropagation). Second, the learning rule is local due to the sum-separability property of the energy function in physical systems. We also touch on this in the next section.

The EP algorithm can be implemented on digital hardware using a discrete-time implementation of the state dynamics (Ji and Gross, 2020). However, this is a slow process as it involves long phases of numerical optimization before convergence, in essence similar to a simulation. As the EP algorithm is inherently a continuous-time optimization method, this motivates the exploration of analog implementations.

Several works have proposed analog implementations of EP in the context of Hopfield networks (Foroushani et al., 2020; Zoppo et al., 2020). A recent study showed that a class of analog neural networks called non-linear resistive networks are EBMs and possess an energy function whose stationary point is the steady-state solution of the analog circuit (Kendall et al., 2020). This result provides theoretical ground for implementing an end-to-end hardware that performs inference and training on the same circuit. Consequently, it serves as the inspiration on which our framework is based.

### 2.4. Example: A simple regression model

**Figure 2:**
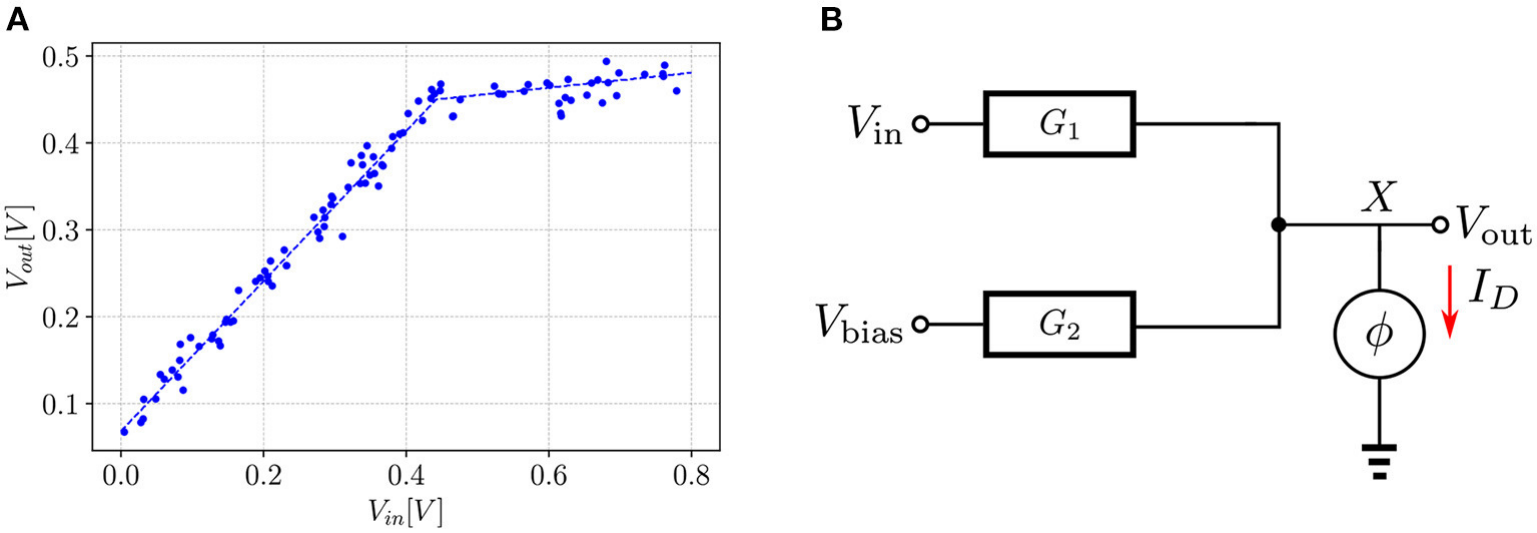
Example of a learning task in EBM: **(A)** Dataset which the model learns. Each dot represents a sample point from the dataset. The dashed line represents the regression line. **(B)** Circuit model that learns the regression line. ϕ is nonlinear function that relates the voltage to the current. The red arrow shows the direction of the current through the non-linear element.

In this section, we elaborate on the learning process of an EBM by demonstrating the construction of the energy function and how the training process shapes the energy surface. To visualize the actual surface rather than its projection to a lower dimension, we construct a contrived example of a simple regression model that can learn the dataset shown in Figure 2A. This shape was chosen for two reasons: (1) It can be implemented in real circuit components, such as a diode. (2) The pseudo-power of the circuit can be easily calculated.

Figure 2B shows the schematic of the model. The input is provided through *V*_in_ and the output is taken from node *X*. The second input is held at a fixed voltage *V*_bias_. Connection to the output node is made through series conductances *G*_1_ and *G*_2_. A non-linear element is attached to node *X*. It has two regions of operation: it behaves as an open-circuit when *V*_out_<*V*_TH_ and as a voltage source, *V*_TH_, in series with a resistance *r*_*on*_ when *V*_out_>*V*_TH_.

The “energy function” of non-linear resistive networks is a quantity called the total pseudo-power of the circuit (Johnson, 2010), and its existence can be derived directly from Kirchhoff's laws. Moreover, this energy function has the sum-separability property: the total pseudo-power of the circuit is the sum of the pseudo-powers of its individual elements. It can be shown that the pseudo-power of a two-terminal element with terminals *i* and *j*, characterized by a well-defined and continuous current-voltage characteristic *I*_*ij*_ = ϕ_*ij*_(Δ*V*_*ij*_) is given by


(4) $$\begin{array}{l} p_{ij}\left(\Delta V_{ij}\right)=\overset{\Delta V_{ij}}{\underset{0}{\int }}\phi_{ij}\left(v\right)\text{d}v. \end{array}$$

The quantity *p*_*ij*_(Δ*V*_*ij*_) has the physical dimensions of power, being a product of a voltage and a current.

With the above definition, and the sum-separability property of the energy function, the total pseudo-power of the circuit shown in Figure 2B can now be calculated.


$$\begin{array}{l} \begin{array}{c} \left[b\right]E=G_{1}\overset{V_{\text{out}}-V_{\text{in}}}{\underset{0}{\int }}v\text{d}v+G_{2}\overset{V_{\text{out}}-V_{\text{bias}}}{\underset{0}{\int }}v\text{d}v\\ +\overset{V_{\text{out}}}{\underset{0}{\int }}\max\left(0,\frac{v-V_{\mathrm{TH}}}{r_{\mathrm{on}}}\right)\text{d}v \end{array} \end{array}$$

(5) $$\begin{array}{l} \begin{array}{c} \left[b\right]E=G_{1}\frac{{\left(V_{\text{out}}-V_{\text{in}}\right)}^{2}}{2}+G_{1}\frac{{\left(V_{\text{out}}-V_{\text{bias}}\right)}^{2}}{2}\\ +\frac{{\left(\max\left(V_{\text{out}},V_{\text{TH}}\right)-V_{\text{TH}}\right)}^{2}}{2\cdot r_{\text{on}}} \end{array} \end{array}$$

Given ***θ*** = (*G*_1_, *G*_2_) and *V*_in_, the energy function associates with each state *s* = {*V*_out_} a real number *E*(***θ***, *V*_*in*_, *s*). For a given input, the effective state $s^{⋆}=s\left(\theta ,V_{\text{in}}\right)$ is the state *s* that minimizes the energy function; i.e., *s*^⋆^ such that $\frac{\partial E}{\partial s}\left(\theta ,V_{in},s^{⋆}\right)=0$. In a non-linear resistive network with two-terminal components, this equilibrium state is exactly the steady state of the circuit imposed by Kirchhoff's laws.

**Figure 3:**
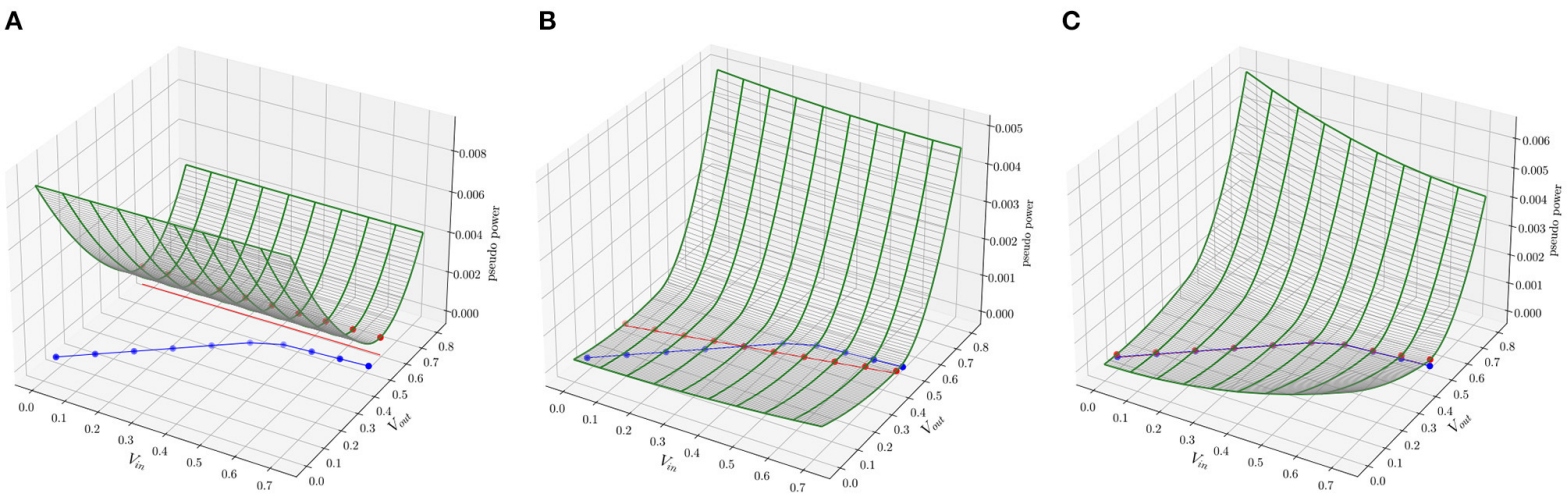
Evolution of the energy surface during training for the task in Figure 2. The goal of training is to mold the energy surface such that the minima are associated with points defined on the blue curve. **(A)** In the beginning of training, the minima of the energy surface are associated with points defined on the red curve, which depend on the random initialization of the parameters of the model. **(B)** After 5 epochs, the shape of the energy surface has changed to create minima closer to the desired points. **(C)** After 10 epochs, the minima are over the desired points.

Figure 3 shows three snapshots of the energy surface during the course of training. In the leftmost plot, initializing the network with random conductances defines an energy surface that associates low energy with states (depicted with red dots on the *xy*-plane) different from the desired ones (depicted with blue dots). The goal of training is to adjust the conductance values to generate an energy surface that associates low energy with the desired states. In some cases, this may not be possible if the energy function is not expressive enough. For instance, there is no set of conductance values that can mold the energy surface to produce equilibrium points defined along a parabola for the circuit in Figure 2B. However, as shown in the rightmost plot, it is possible to obtain a set of conductance values that shape the energy function to produce the regression line in Figure 2A.

## 3. Exploratory framework

**Figure 4:**
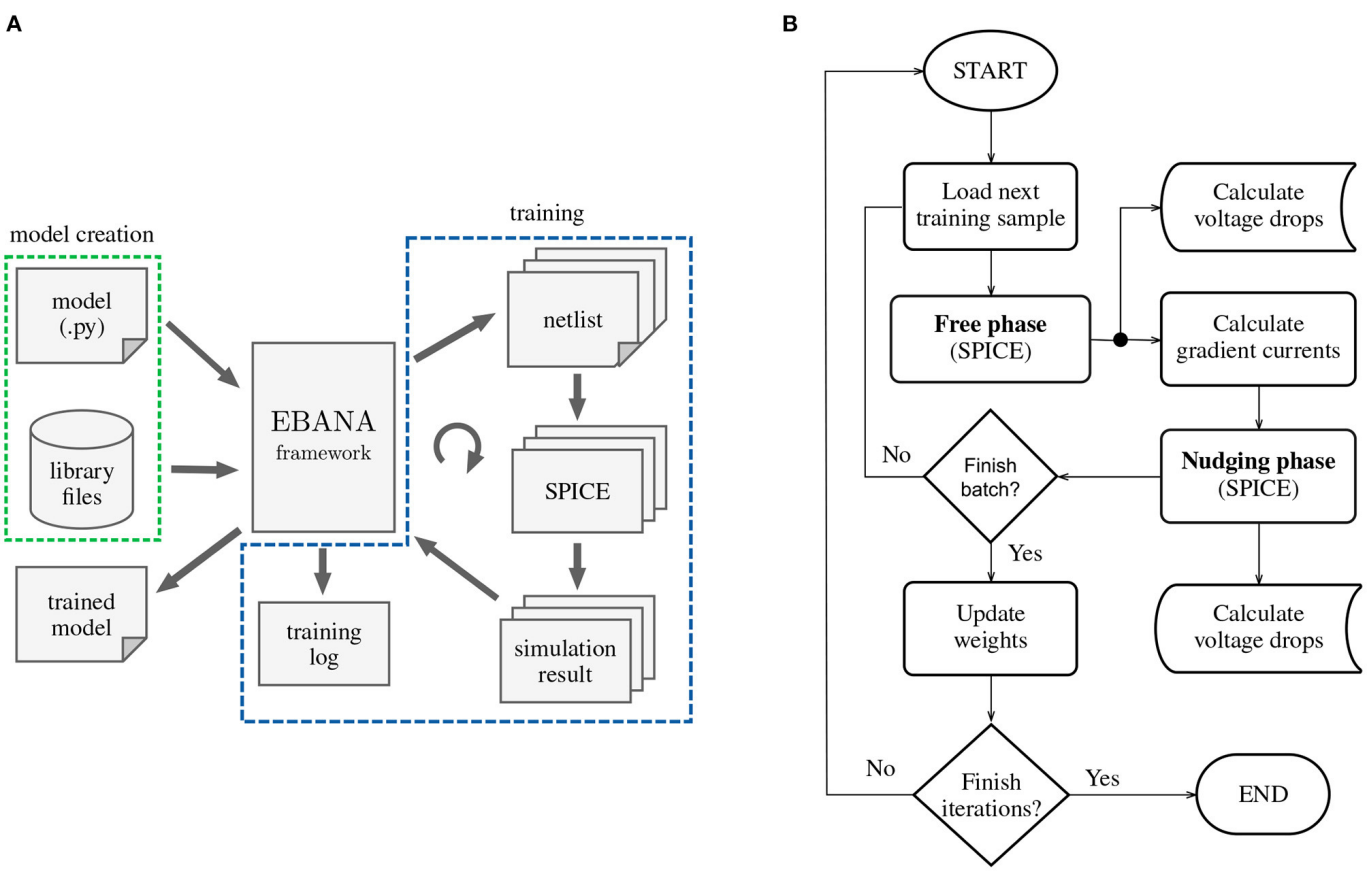
High level organization of EBANA: **(A)** Topologies such as the one shown in Figure 5 can be easily composed with a few lines of python code. Together with the library files (SPICE models, subcircuits, etc.), the EBANA framework dispatches the generated netlist in batches to the SPICE simulator. The EBANA framework then executes the EP algorithm on the result of the simulation, as well as keep track of the evolution of important parameters such as voltages and currents. These can later be studied to reveal important trends that can help in fine-tuning the learning process, as well as in the evaluation of the power consumption of the system. **(B)** The implementation of the fit method follows closely that of any traditional ML training loop. The difference here is that a SPICE simulator is used, and the parameters of the network are updated based on the data points at only two time steps.

Our framework, EBANA, provides a comprehensive solution for designing and training neural networks in the analog domain. The architecture is comprised of two main parts: one for defining the network model, and the other for training in the analog domain. A high level view is shown in Figure 4A.

The interface to EBANA is Python, leveraging its rich ecosystem of libraries for data processing and data analysis. With the exception of circuit simulation, all operations, including netlist generation, gradient computation, and weight updates, are performed in Python.

We employ a SPICE simulator for realistic simulation of the circuit dynamics, with PySpice^2^ serving as the bridge between Python and the simulator. PySpice supports two of the most widely used open-source SPICE simulators, Ngspice^3^ and Xyce.^4^ Ngspice is readily available on almost all popular operating systems and is the default simulator for EBANA. Xyce supports large-scale parallel computing platforms and is attractive for complex deep learning problems. The choice between the two simulators can be made by simply setting a global variable. It's worth noting that the vanilla build of Ngspice has a subcircuit node limit of 1,000, whereas Xyce does not have this limitation, though it requires compiling the source code.

### 3.1. Network structure

**Figure 5:**
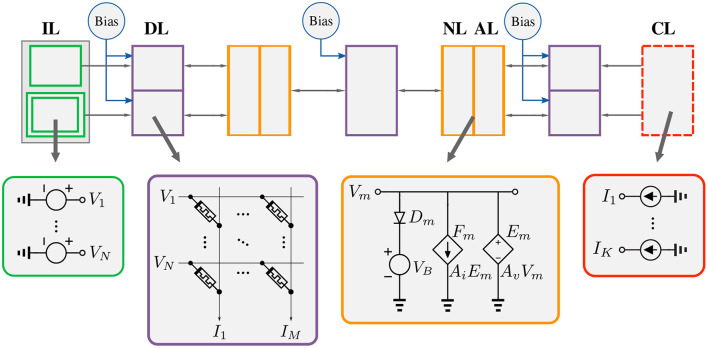
Analog neural network in the EP framework. IL, Input Layer; DL, Dense Layer; NL, Non-linearity Layer; AL, Amplification Layer; CL, Current Layer.

The process of designing and training a model in our framework starts with defining the model. A typical structure of an analog neural network that can be trained with the EP framework is shown in Figure 5. It consists of an input layer, several hidden layers, and an output layer. It looks similar to a regular neural network that can be trained by the backpropagation algorithm except for two major differences. First, the layers can influence each other bidirectionally; i.e., the information is not processed step-wise from inputs to outputs but in a global way. Second, the output nodes are linked to current sources which serve to inject loss gradient signals during training.

### 3.2. Creating a model

**Figure 6:**
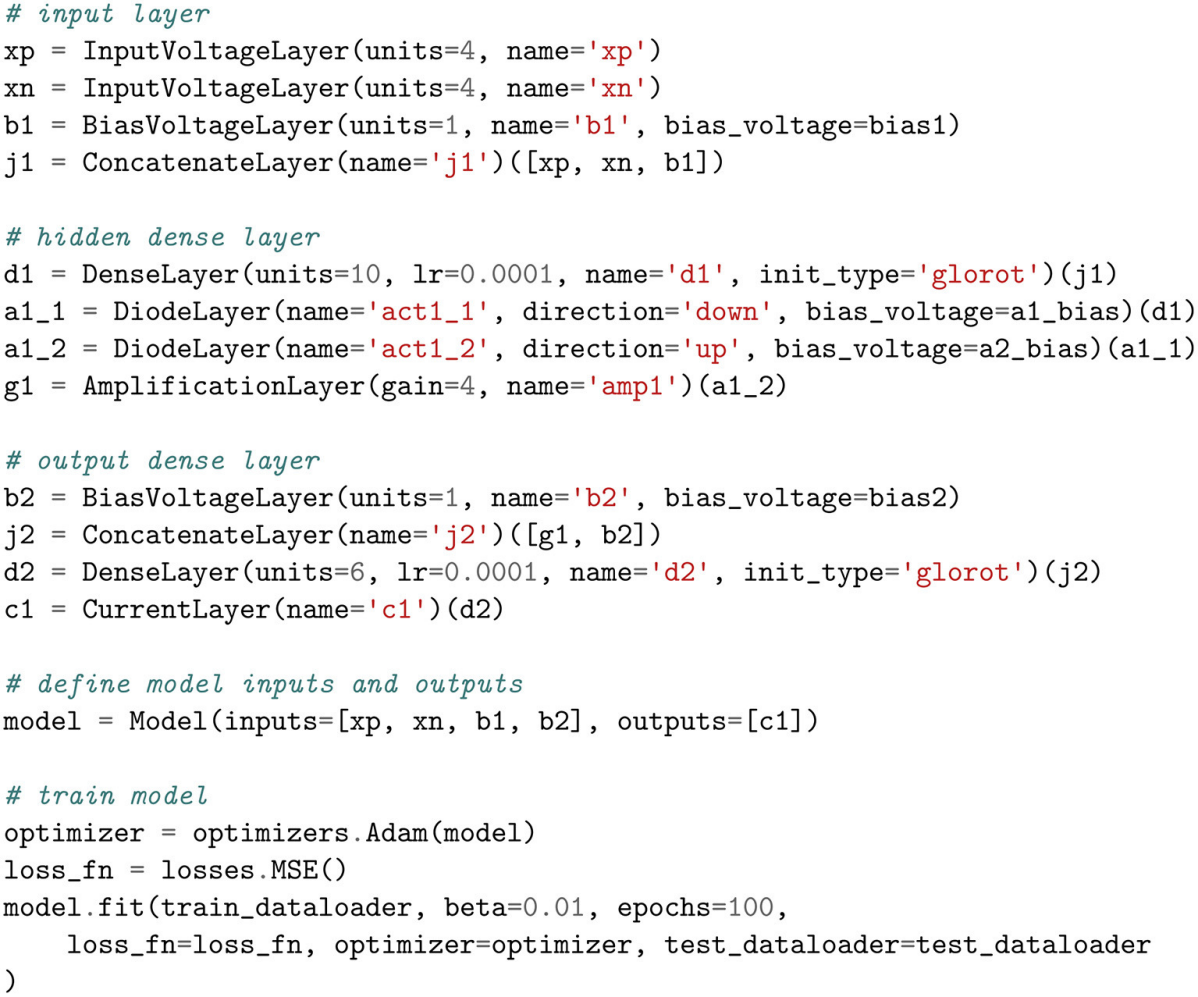
Example of a model in the EBANA framework (Iris model).

Layers, which are essentially subcircuits in analog circuits, form the core data structure of our framework. They are expressed as Python classes whose constructors create and initialize the pin connections, and whose call methods build the netlist. The process of creating a model is heavily inspired by Keras's functional API due to its flexibility at composing layers in a non-linear fashion. In this manner, the user is able to construct models with multiple inputs/outputs, share layers, combine layers, disable layers, and much more. An example of this is given in Figure 6, which follows the structure shown in Figure 5. In the following subsections, we provide details on only those layers that have a unique interpretation in the analog domain.

#### 3.2.1. Input layer

This defines the number of inputs to the circuit, which are typically represented by voltage sources. Generally, the input layer is defined according to the dataset. However, the input layer can be defined slightly differently in the analog domain.

- First, since the weights are implemented by resistors, and resistances cannot be negative, a second set of inputs with the opposite polarity of the voltages defined in the dataset is added to the input layer. This accounts for negative weights and effectively doubles the number of inputs and storage elements. This idea (a voltage layer that is the same but opposite in polarity to another one) is depicted with two green rectangles in Figure 5. Note that this can avoided by setting the reference voltage to some value other than 0. In this way, all voltages less than the reference voltage are considered negative. However, this requires shifting all other voltage nodes in the circuit by the new reference value.
- Second, in typical software-based frameworks, the bias, when used, is implicitly set to 1. However, since circuits can work with a wide range of voltages, setting the bias voltage to values other than 1 is necessary. Hence, we provide the option to independently set the bias voltage in each layer. Note that it is also possible to learn the bias voltages.

#### 3.2.2. Weight layers

Two kinds of weight layers are defined in the framework: the Dense layer and the LocallyConnected2D layer. The Dense class is the implementation of the fully-connected layer, which means that each neuron of the layer is connected to every neuron of its preceding layer. This connectivity pattern can be easily implemented in crossbar arrays by simply connecting each row of the crossbar array to all columns of the previous layer's crossbar array.

The implementation of fully-connected layers is straightforward, but implementing convolutional layers in the analog domain is challenging as the filters are connected to a local region of the previous layer. To achieve this connectivity pattern, a more complex wiring is necessary in crossbar arrays. While it can still be done by shifting the inputs and temporarily storing them in buffers, the dot product operation becomes a non-constant time process (Boser et al., 1991). To overcome this, we have implemented a variant of the convolutional layer called the LocallyConnected2D layer, where the dot product operation is between a section of the input matrix and the filter, with a different filter used for each subregion of the input, avoiding the weight sharing issue.

Another issue that is specific to ANNs is the weight initialization problem. Neural networks are very sensitive to the initial weights, and thus selecting an appropriate weight initialization strategy is critical to stabilize the training process. As a result, a lot of research has gone into finding optimal weight initialization strategies (Li et al., 2020). However, since conductances cannot be negative, these methods cannot be applied directly. Hence, although we provide a default range, some experimentation is advised.

#### 3.2.3. Non-linearity

The non-linearity layer is implemented with a diode in series with a voltage source. We provide two kinds of diodes: a regular diode and a MOS diode. They have the following options:

- **Diode orientation** (direction): This specifies the orientation of the anode and cathode of the diode with respect to the voltage source.
- **Bias voltage** (bias): By choosing a bias value other than zero, we can change the voltage at which the diode saturates, and therefore alter the shape of the non-linearity.
- **SPICE model** (model): This is a text description that is passed to the SPICE simulator that defines the behavior of the diode.

#### 3.2.4. Amplification layer

Unlike the dense layer used in libraries like Tensorflow where the output is the weighted sum of the inputs, the output of a resistive crossbar array is the weighted mean of the inputs. This has the effect of reducing the dynamic range of the signal. As a result, amplifiers are needed to restore the dynamic range of the signal as it propagates between the input and output layers.

The amplification layer is implemented with ideal behavioral sources. It boosts the voltages in the forward direction by a factor of *A* and the currents in the reverse direction by a factor of $\frac{1}{A}$. Without the reverse current, the circuit reduces to a signal-flow model where the outputs no longer affect the inputs and the algorithm fails. Furthermore, the $\frac{1}{A}$ factor is to ensure that the gain of the amplifier does affect the magnitude of the reverse current; i.e., a load connected to the output node of the amplifier has the same effect at the input node as a load connected directly to the input node.

#### 3.2.5. Current source layer

This layer simply adds current sources at each output node to inject current into the network during the nudging phase. It is implemented with ideal current sources. During the forward phase, the current sources are set to 0.

### 3.3. Training

The training process that is implemented by the fit method is illustrated in Figure 4B.

#### 3.3.1. Weight gradient calculation

The current gradients are calculated according to the chosen loss function. For instance, in the case of the mean squared-error (MSE), the loss is given by $C\left({Ŷ}_{k},Y_{k}\right)=\frac{1}{2}{\left({Ŷ}_{k}-Y_{k}\right)}^{2}$, where *k* is the index of output node, Ŷ_*k*_ is the output of the node, and *Y*_*k*_ is the target value. Other loss functions such as the cross-entropy loss are also available.

The current that is injected into output node *k* is some multiple β of the derivative of the loss with respect to that node: i.e., $-\beta \frac{\partial C}{\partial {Ŷ}_{k}}$. The negative sign is for gradient descent.

To address the constraint of non-negative weights, the number of output nodes are doubled. That is, the output node Ŷ_*k*_ is represented as the difference between two nodes: ${Ŷ}_{k}={Ŷ}_{k}^{+}-{Ŷ}_{k}^{-}$. The currents, $I_{k}^{+}$ and $I_{k}^{-}$, that are to be injected into $Y_{k}^{+}$ and $Y_{k}^{-}$, respectively, are:


(6) $$\begin{array}{l} \begin{array}{c} I_{k}^{+}=-\beta \frac{\partial C}{\partial {Ŷ}_{k}^{+}}=\beta \left({Ŷ}_{k}+{Ŷ}_{k}^{-}-Y_{k}^{+}\right)\\ I_{k}^{-}=-\beta \frac{\partial C}{\partial {Ŷ}_{k}^{-}}=\beta \left({Ŷ}_{k}^{+}-{Ŷ}_{k}^{-}-Y_{k}\right) \end{array} \end{array}$$

#### 3.3.2. Weight update

During the free phase, the current sources at the output nodes are set to 0. The inputs are applied and circuit is allowed to settle. We then collect the node voltage $V^{0}=\left(V_{1}^{0},\ldots ,V_{N}^{0}\right)$ and calculate the voltage drop $\Delta V_{ij}^{0}$ across each conductance.

In the nudging phase, the current given by Equation (6) is injected into each output node. After the circuit settles, we collect the node voltages $V^{\beta }=\left(V_{1}^{\beta },\ldots ,V_{N}^{\beta }\right)$ and calculate the voltage drop $\Delta V_{ij}^{\beta }$ across each conductance once again. We then update each conductance according to the equation below (Kendall et al., 2020).


(7) $$\begin{array}{l} G_{ij}\leftarrow G_{ij}-\frac{\alpha }{\beta }\left[{\left(\Delta V_{ij}^{\beta }\right)}^{2}-{\left(\Delta V_{ij}^{0}\right)}^{2}\right] \end{array}$$

where α is the learning rate.

The weight update rule as defined by (7) is one of the options available in the optimizer class, and is defined under the name SGD (stochastic gradient descent). Other weight update mechanisms such as SGDMomentum (stochastic gradient descent with momentum) and ADAM are also available.

The momentum method can speed up training in regions of the solution space that are nearly flat by adding history to the conductance update equation based on the gradient encountered in the previous updates. The ADAM update rule takes this idea one step further by adapting a learning rate for each conductance, thereby dulling the influence of conductances with higher gradients and boosting those with smaller gradients.

During the early stages of training when the conductances are rapidly changing, the value of the update term $\frac{\alpha }{\beta }\left[{\left(\Delta V_{ij}^{\beta }\right)}^{2}-{\left(\Delta V_{ij}^{0}\right)}^{2}\right]$ can sometimes be larger than *G*_*ij*_. This will result in numerical instability as some conductances are now negative. To address this issue, all conductance values that fall below a certain threshold are clipped to that threshold.

### 3.4. Parallelism

Training with EP requires performing the free phase and nudging phase, after which the conductances are updated. Both of these phases are done sequentially in SPICE, and are the critical path in the pipeline. While SPICE simulations are always going to be time consuming, the overall simulation time can be reduced by running many simulations in parallel. This is achieved by noting that all the samples in a mini-batch are independent and, therefore, could be simulated independently. As a result, the simulation time could in theory be limited only by the time it takes to simulate a single sample in a batch.

## 4. Evaluation

In this section, we evaluate our framework focusing on three aspects: correctness, extensibility, and performance.

### 4.1. Illustrative example: Learning the iris dataset

As a first step in the evaluation, we built a model that could learn the Iris dataset.^5^ This example is a well-known problem of moderate complexity, containing 150 samples, with 4 input variables and 1 output variable that takes values 3 values.

Two preprocessing steps are needed before the data is ready for training. First, the input variables have to be normalized. Second, we associate with each unique output value a 3-bit one-hot encoded value. Hence, after the preprocessing step, the dataset has 4 inputs and 3 outputs.

We constructed a model with 1 input layer, 1 hidden layer, and 1 output layer, as shown in Figure 6. The input layer has 9 nodes; 4 for the regular inputs, 4 for the inverted set, and 1 for the bias. In the preprocessing step, the data was scaled to take real values in the range [−0.5V, 0.5V] so that it is compatible with modern CMOS process voltages.

The hidden layer was implemented with 10 nodes and the output layer with 6 nodes. The weights were initialized from samples drawn randomly from the range $\left[10^{-7}\text{S},\frac{8\cdot 10^{-8}}{\sqrt{n_{\mathrm{in}}+n_{\mathrm{out}}+1}}\text{S}\right]$, where *n*_in_ is the size of the inputs and *n*_out_ is the number of nodes. The learning rate of both layers was set to 4·10^−4^.

The dataset was split into two parts: 105 samples for training, and 45 samples to evaluate the model on new data while training. The optimizer was set to ADAM and the model was trained for 400 iterations. It achieved an accuracy of 100% on the test dataset. A plot of the loss and accuracy as a function of the number of the training epochs is shown in **Figure 8A1**. This validates the correctness of our framework.

### 4.2. Effect of model parameters on model performance

The non-linearity of activation functions used in deep learning models is crucial for the learning process. Without them, the model reduces to a linear composition of layers. In terms of the energy surface, this means that all the equilibrium points lie along a straight line, preventing the model to capture anything but linear responses. The addition of non-linearities creates a much richer energy surface that greatly enhances the model's capability to learn.

**Figure 7:**
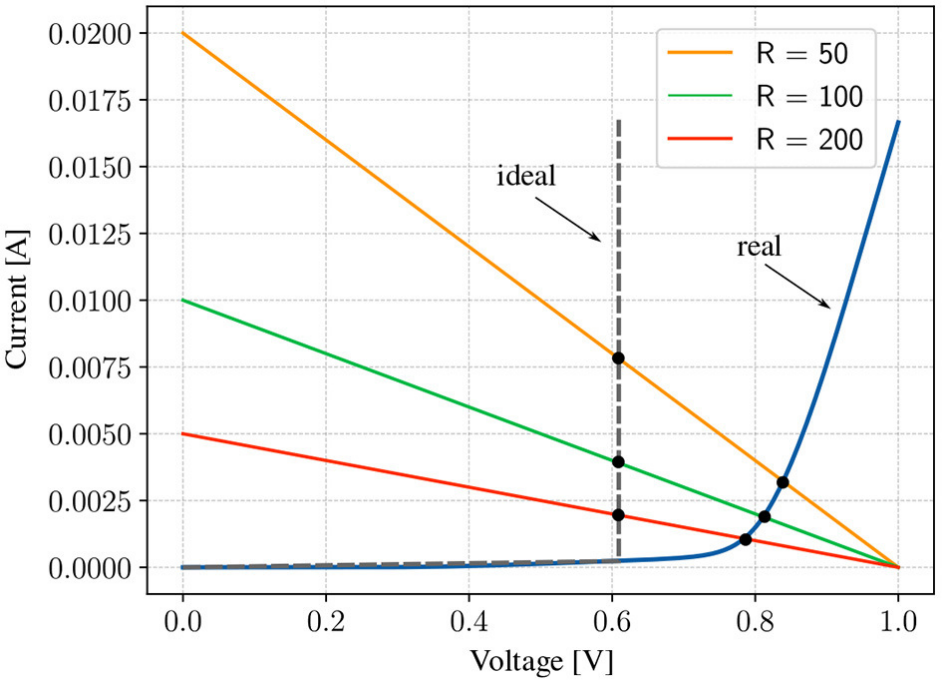
Illustration of the operating point of the circuit as *R*_Thev_ is changed for both an ideal diode and a real diode. In the case of a real diode, the smaller the resistances in the dense layer, the higher is the voltage at which the diodes saturate or the lesser is the non-linearity effect for the same voltage range. Even though we need amplifiers with lower gains, but because the resistances are now smaller, a larger current flows through the circuit, causing the power consumption to go up.

Our model incorporates non-linearity through the non-linear current-voltage (I-V) characteristics of a diode, as depicted in the blue curve in Figure 7. This plot resembles the ReLu function commonly used in deep learning, but with two key differences:

(1) The plot here represents the current-voltage transfer function, not the voltage-voltage transfer function. When the voltages applied in the circuit are below the knee of the diode's I-V curve, the diode draws minimal current, resulting in a nearly linear circuit. Non-linearity only arises when the operating point is above the knee of the curve and the diode begins to draw current, which is the opposite behavior to the ReLu function.
(2) Because of loading effects in the analog domain, the voltage at the output node of the diode is a non-linear function of the entire circuit, not just the layers preceding it. This has two implications: (a) To know the voltage at the output node of the diode, the entire circuit has to be solved. (b) The shape of the non-linearity (or the voltage at which the diode saturates) is affected by the circuit parameters.

We can get some insight into the non-linear behavior of the diode by modeling the circuit around it with a Thevenin voltage (*V*_Thev_) and a Thevenin resistance (*R*_Thev_). In this case, the operating point (Q-point) of the circuit is the intersection of the I-V characteristic of the diode and that of the load line, given by the equation $I_{D}=\frac{V_{\text{Thev}}-V_{D}}{R_{\text{Thev}}}$, where *I*_*D*_ is the current through the diode, and *V*_*D*_ is the voltage across it. In the case of an ideal diode, *V*_*D*_ = const, indicating that the diode saturates at the same voltage irrespective of the value of *R*_Thev_ (similar to how a non-linear function behaves). However, real diodes offer a resistance in series with *R*_Thev_, causing the diode to saturate at different values depending on the size of *R*_Thev_. This complex relation affects the input dynamic range that can be used, the gain needed for the amplifiers, and the overall power consumption of the circuit. Here, we investigate the interplay between these factors on the model performance.

**Figure 8:**
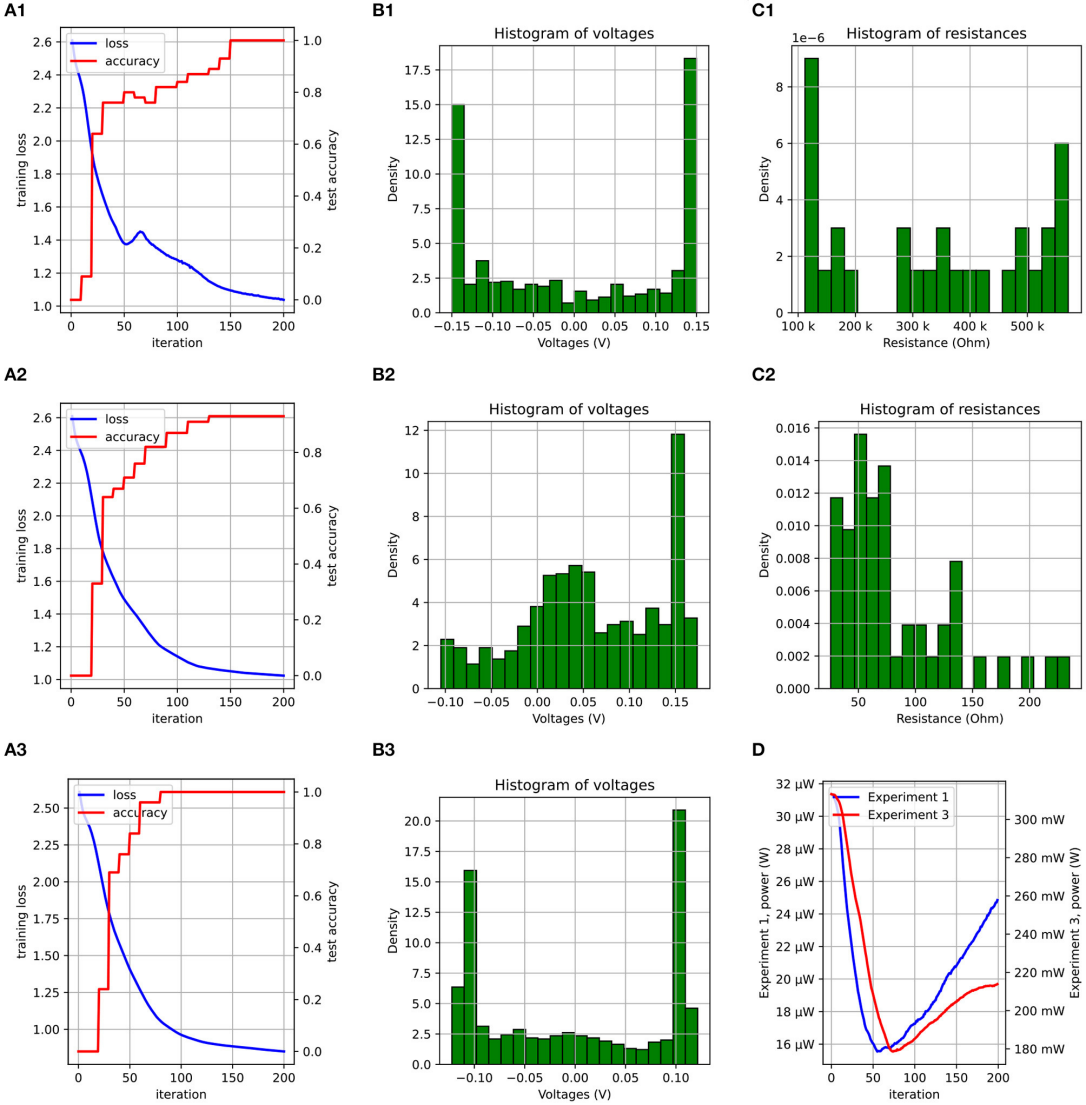
A plot of the loss/accuracy for experiments 1, 2, and 3 is shown in **(A1–A3)**. **(B1–B3)** Show the voltage distribution. **(C1, C2)** Show the resistance distribution of the first dense layer at the end of training. **(D)** Compares the power consumption of the circuit during training for experiments 1 and 3.

Figure 7 shows how the Q-point of the circuit changes as *R*_Thev_ is changed. For a fixed *V*_Thev_>*V*_TH_, increasing the resistance reduces the voltage at which the diode saturates. This reduces the dynamic range of the signal, forcing the use of amplifiers with higher gains. While the actual behavior of the circuit is more complex, this insight equips us with a beacon to search the parameter space for better initial points. To test this hypothesis, we designed an experiment similar to the one in the previous section, but with the conductances multiplied by 10^4^. The distribution of the conductances (or resistances) in the first and second experiments is shown in Figures 8C1, C2, respectively. The values of beta (β) and the learning rates (α) have to be scaled by roughly the same factor. We obtained an accuracy of 93% after 200 epochs (Figure 8A2), compared to 100% in the first (Figure 8A1). The loss in accuracy can explained by the fact that the nonlinearity is weaker in the second experiment due to the smaller resistances in the dense layer. To support the claim that the non-linearity is weaker due to the smaller resistances, bias voltages were applied to allow the diodes to saturate earlier by 0.05V. With this modification, the accuracy improved to 100% (Figure 8A3). The distribution of the voltages at input of the amplifer for the three cases is shown Figures 8B1–B3. The weaker non-linearity in the second experiment resulted in a voltage distribution with a higher density around 0V, as opposed to the other two, where the density is highest around the saturation voltages. Finally, even though the adjustment made to the second experiment improved the accuracy, the power consumption of the circuit is roughly 10^4^ more (Figure 8D).

### 4.3. Mixed-signal application

**Figure 9:**
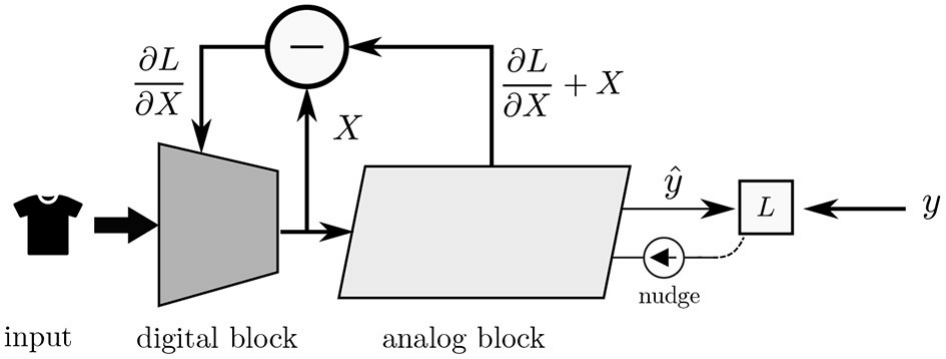
High level view of integrating a digital neural network with an analog neural network based on the EP algorithm. The digital block receives a high-dimensional input, downscales it, and feeds the result to the analog block, which does the bulk of the computation. The analog block “backpropagates” error signals to the digital block such that the parameters of both blocks are adjusted in the direction that reduces the energy of the system.

In this section, we explore the possibility of integrating a digital component to our analog model, in a so-called mixed-signal design. The idea is depicted in Figure 9. Here, the inputs, for example a high-dimensional image, is introduced to the digital block, preprocesses the data and embeds the input in a lower-dimensional space before passing it to the analog block. The reason for doing this is the following: A convolutional layer reuses the same input data and a relatively small number of weights over many sequential operations. Meanwhile, a fully connected layer typically involves a much larger number of weights with no input data reuse. Furthermore, as convolutional operations tend to be computation-bound, while fully connected layers are bounded by the memory bandwidth, it is thus advantageous to implement convolutional layers in digital and fully-connected layers in analog.

The proposed mixed-signal implementation was evaluated on the Fashion-MNIST dataset. Fashion-MNIST is a popular machine learning benchmark task that improves on MNIST by introducing a harder problem, increasing the diversity of testing sets, and more accurately representing a modern computer vision task.

In our approach, Keras was used to play the role of the digital block while EBANA acted as the analog block. The process of training the mixed-signal system is as follows:

1. We built a model with the parameters shown in Table 1 and trained it for 20 epochs. We achieved an accuracy of 90% on the test dataset.
2. Using the trained model, we passed the entire Fashion-MNIST dataset through the layers of the model, and collected the result from the Flatten layer. This step represents embedding the input vector from a dimension of 784 into dimension of 150.
3. We then trained an analog model similar to the one in Figure 6 but with 100 nodes in the hidden layer. We stopped training after 1 epoch after achieving an accuracy of 85% on the test dataset using the cross-entropy loss.
4. Using the trained analog model, we then trained the inputs (i.e., gradient descent on the input) until we achieved an accuracy of 100%. This new set of inputs represents the inputs that the analog block expects from the digital block if the accuracy is to be improved.
5. Back in Keras, a new model was trained using the original dataset but with the objective of producing the trained inputs from the previous step. We then repeated steps 2 and 3. The accuracy improved by 3%.

**Table 1:** Keras model for Fashion-MNIST dataset.

**Layer**	**Parameters**
Conv2D	Filters = 8, kernel_size = 5, activation = “relu”
MaxPooling2D	Pool_size = 2
Conv2D	Filters = 8, kernel_size = 5, activation = “relu”
MaxPooling2D	Pool_size = 2
Flatten	–
Dropout	*p* = 0.15
BatchNormalization	-
Dense	Units = 512, activation = “relu”
Dense	Units = 10, activation = “softmax”

The result of this experiment shows that we can “backpropagate” through the analog layer, opening the possibility of a full-fledged mixed-signal implementation where the analog block benefits from the preprocessing opportunities available in the digital domain.

### 4.4. Extensibility

Even though it is possible to design fully functional ANNs with the EBANA framework, we provide sufficient system encapsulation and model extensibility to meet the individual requirements of incorporating new models and extending the functionality of the framework, beyond Energy-Based Models. This includes adding new layers, defining new loss functions, changing the training loop, and much more. The only constraint in defining new components is that they must be constructed of linear and non-linear dipoles to ensure stability, as stated by Johnson (2010).

**Figure 10:**
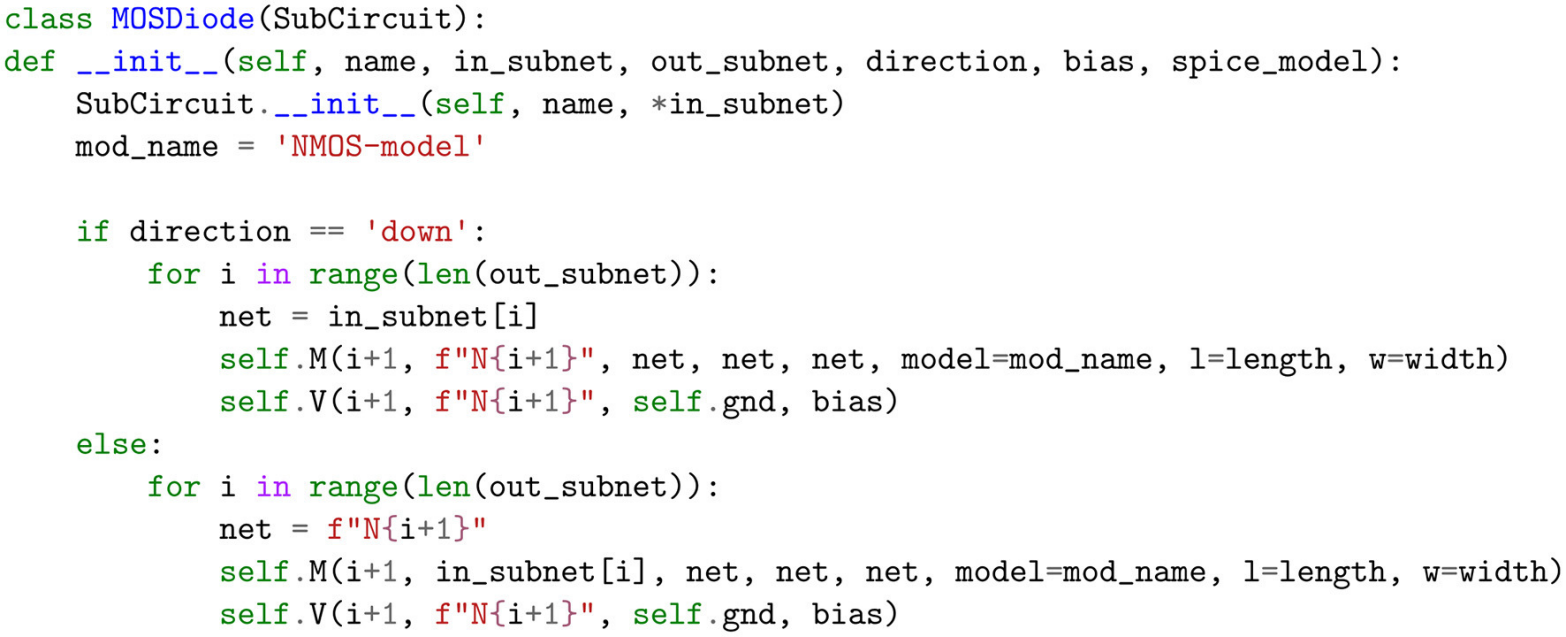
Example of defining a new kind of layer.

To demonstrate the extensibility capabilities of our framework, we consider the example shown in Figure 10. Here, we show that by subclassing the SubCircuit class, and with a just a few lines of code, a new kind of non-linearity can be defined using MOSFET transistors and voltages sources.

Our library is modularized to easily plug in or swap out components. For instance, to investigate the circuit behavior with this new kind of non-linearity, all we have to do is replace the DiodeLayer in Figure 6 with MOSDiode layer in Figure 10 and rerun the simulation. Moreover, while the circuit in Figure 5 is setup for training, it can be easily converted to one that measures the compatibility of an input-output pair by simply swapping the current layer with a voltage layer that represents the output.

### 4.5. Performance

To evaluate the performance of the simulator, two experiments were conducted. The first experiment was conducted on the Iris model with the goal of measuring the speed-up gained through parallelism. We fixed the number of samples in the mini-batch and ran the simulation for the same number of epochs on a single thread, followed by two, and then four. While the speed-up factor was indeed almost doubled when the thread count was increased from 1 to 2, doubling the thread count further resulted in just 1.5x increase in speed. Due to the resulting circuit being relatively simple, and the small batch size, the overhead of starting new processes for every batch is a non-trivial percentage of the overall simulation time. However, this would not be a problem for experiments with reasonably large datasets.

For the second experiment, we wanted to measure the simulation performance as a function of problem complexity. To this end, we considered 3 datasets; xor, iris, and wine.^6^ To obtain an estimate for the complexity of the circuit, we counted the number of nodes only in those models that achieved an accuracy greater than 95% on the test dataset. This is due to the fact that the bias-variance trade-off is a property of the model size.

The circuits were simulated and the average simulation time in seconds is recorded in Table 2. For a measure of the intrinsic speed of the simulator, a column with a calculated property *K* is added. The property is calculated according to Equation (8) and takes into account the simulation time *T*, the number of allocated threads *P*, the number of nodes in the generated circuit *N*, the number of epochs *E*, and the size of the training dataset *D*. We can see from Table 2 that *K* is about the same for the two examples whose simulation time is not dominated by the overhead of starting the SPICE simulator. We expect this to hold true for larger datasets.

**Table 2:** DC simulation time as a function of circuit size and training dataset size.

**Datasets**	**I/O units**	**Circuit nodes (*N*)**	**Dataset size (*D*)**	**Epochs (*E*)**	**Time (*T*)**	**Threads (*P*)**	**K(10^−4^)**
Xor	5/2	16	4	85	14 s	1	25.74
Iris	9/6	56	105	155	182 s	4	7.99
Wine	25/4	111	5,000	2	217 s	4	7.92

Training for all the experiments was carried out in a laptop with an Intel i7-6700HQ CPU and 32 GB of RAM.


(7) $$\begin{array}{l} K=\frac{T\cdot P}{N\cdot E\cdot D} \end{array}$$

## 5. Conclusion

In this paper, we presented an open-source unified, modularized, and extensible framework called EBANA, that can be used to easily build, train, and validate analog neural networks. By using Python as the interface language, with a syntax similar to Keras, we're able to hide the complexity of the underlying analog simulations and offer researchers in neuroscience and machine learning a conceptual and practical framework to experiment with and explore the various tradeoffs that exist in the design space.

EBANA does not only include the building blocks required for the design of EBMs (i.e., IL, DL, NL, and CL layers); it also maintains sufficient modularity and extensibility to easily incorporate new concepts, electrical and technological models. For example, adding a new non-linear layer requires less than 15 lines of code. New learning concepts beyond EBM can also be easily implemented, as illustrated with the co-training of an EBM with a conventional CNN that uses the backpropagation algorithm. Finally, EBANA has a graph-based data structure that facilitates the composition of networks with a great deal of flexibility. All of these features enable the implementation of a broad range of supervised machine learning tasks in EBANA, and not just those with linear topologies.

While EBANA is already fully functional and can reduce by orders of magnitude the effort required to analyze new analog neural networks, more features and functionalities will be added in future iterations, including a suite of hardware blocks in nanometric technologies for proper evaluation of the energy consumption of the system. At the moment, the framework supports only the open-source simulators Ngspice and Xyce, which introduce some artificial limitations: The default distribution of Ngspice places a limit of 1,000 nodes on the size of subcircuits. This is not an issue for Xyce, but it is not always as readily available. We plan to add support for commercially available simulators such as Specter and Hspice. We also plan on improving the training speed by optimizing the training loop and avoid generating a new netlist for every simulation. This can result in massive speedups, both in Python (where the netlist is generated) and the SPICE simulator which builds a conductance matrix every time it is presented with a new netlist. Finally, we plan to add methods for distributed training over multiple machines.

## Data availability statement

Publicly available datasets were analyzed in this study. This data can be found at: MNIST-Fashion dataset: https://github.com/zalandoresearch/fashion-mnist, Iris dataset: https://archive.ics.uci.edu/ml/datasets/iris, Wine dataset: https://archive.ics.uci.edu/ml/datasets/wine.

## Author contributions

All authors listed have made a substantial, direct, and intellectual contribution to the work and approved it for publication.

## Footnotes

^1^ https://keras.io/guides/functional_api/

^2^ https://pypi.org/project/PySpice/

^3^ http://ngspice.sourceforge.net/

^4^ https://xyce.sandia.gov/

^5^ https://archive.ics.uci.edu/ml/datasets/iris

^6^ https://archive.ics.uci.edu/ml/datasets/wine
